# Supplementary figures and images for: Bidirectional promoter activity from expression cassettes can drive off-target repression of neighboring gene translation
Source: eLife. 2022 Dec 12;11:e81086. doi: 10.7554/eLife.81086 (PMC9754628; doi:10.7554/eLife.81086)

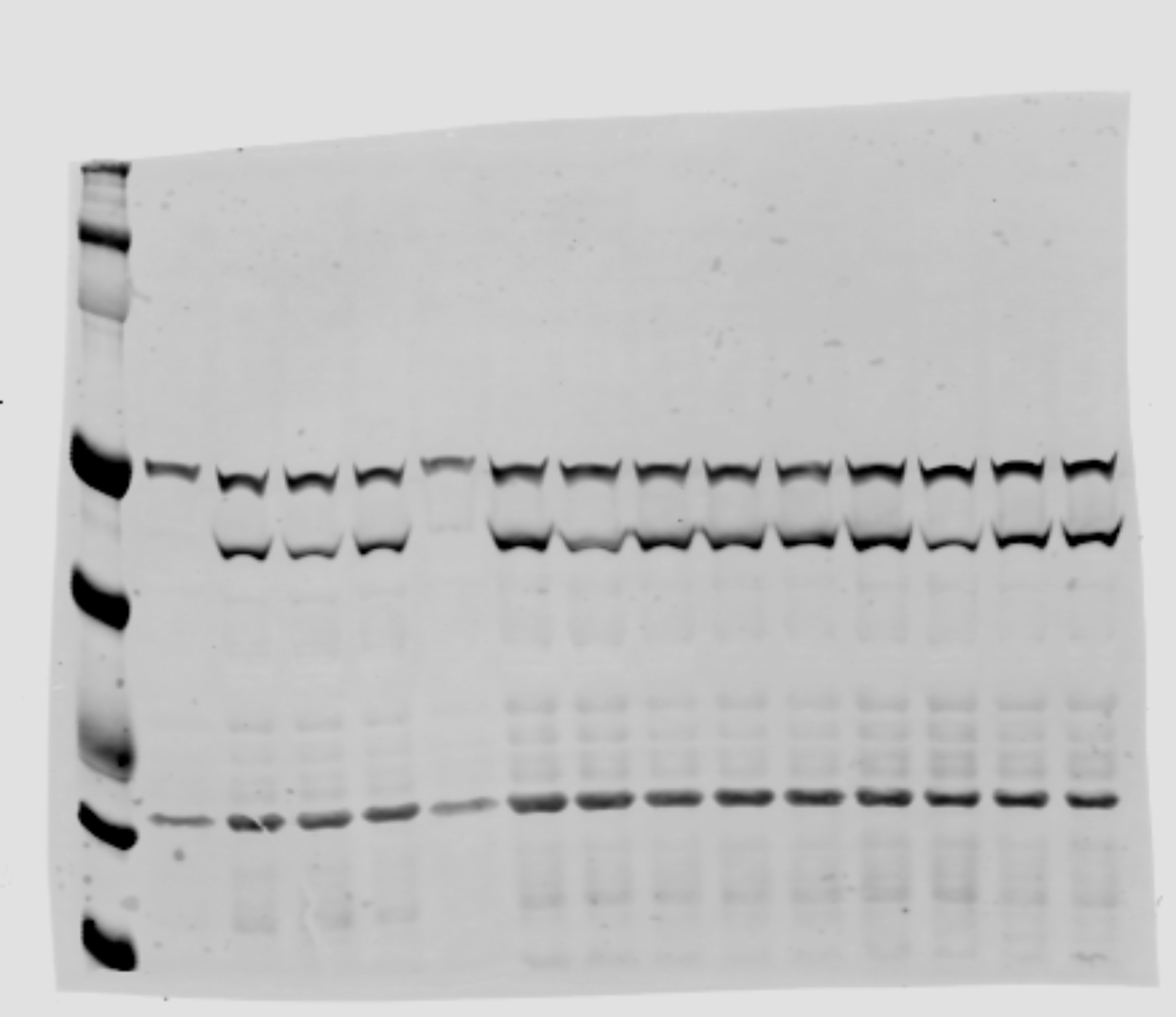

Supplement: Figure 1—figure supplement 1—source data 1. [file elife-81086-fig1-figsupp1-data1.zip › Figure 1 - figure supplement 1 - Source Data 1/Figure 1 - Source Data 2 - unlabeled.tif]

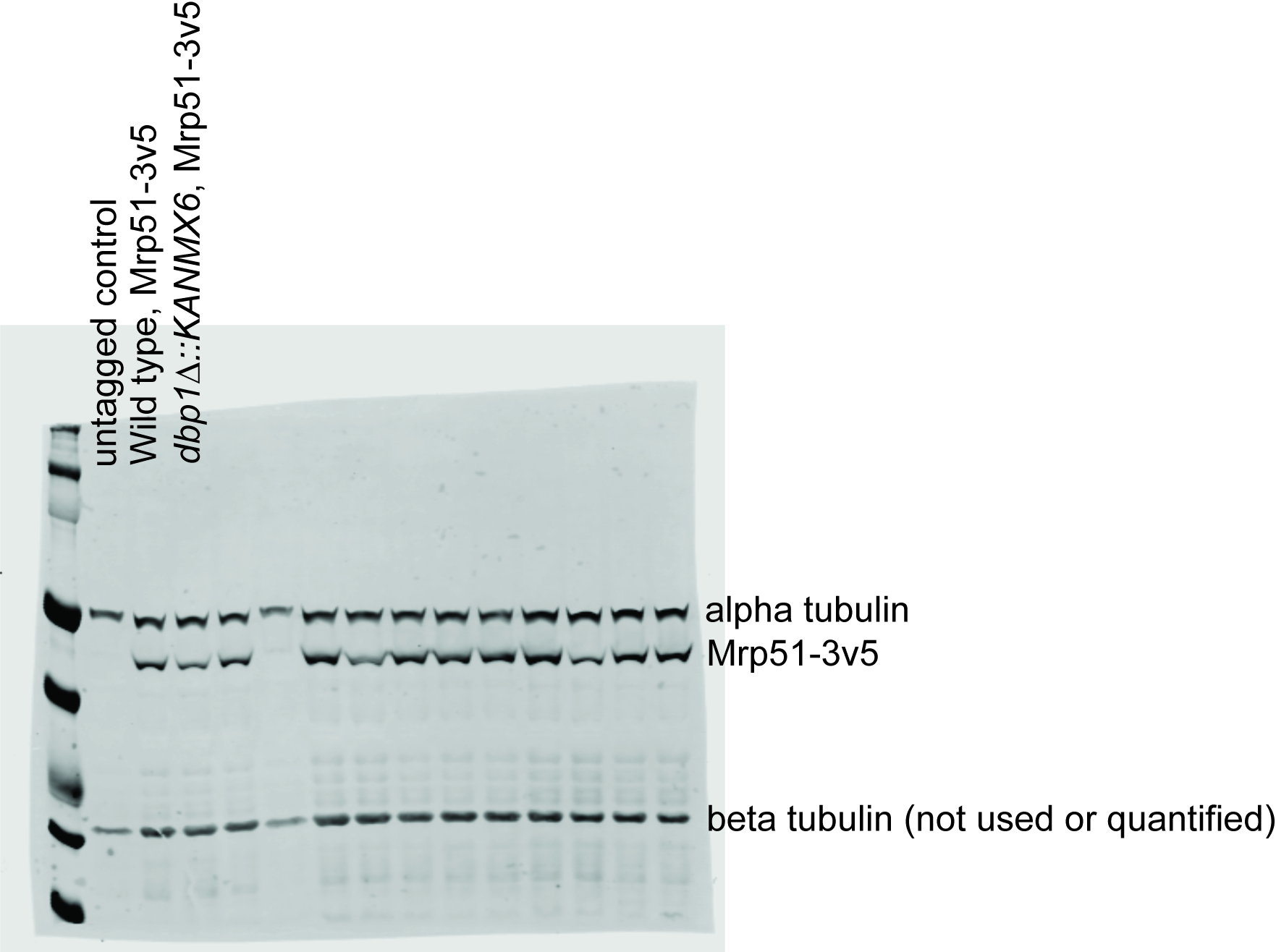

Supplement: Figure 1—figure supplement 1—source data 1. [file elife-81086-fig1-figsupp1-data1.zip › Figure 1 - figure supplement 1 - Source Data 1/Figure 1 - Source Data 2 - labeled.tif]

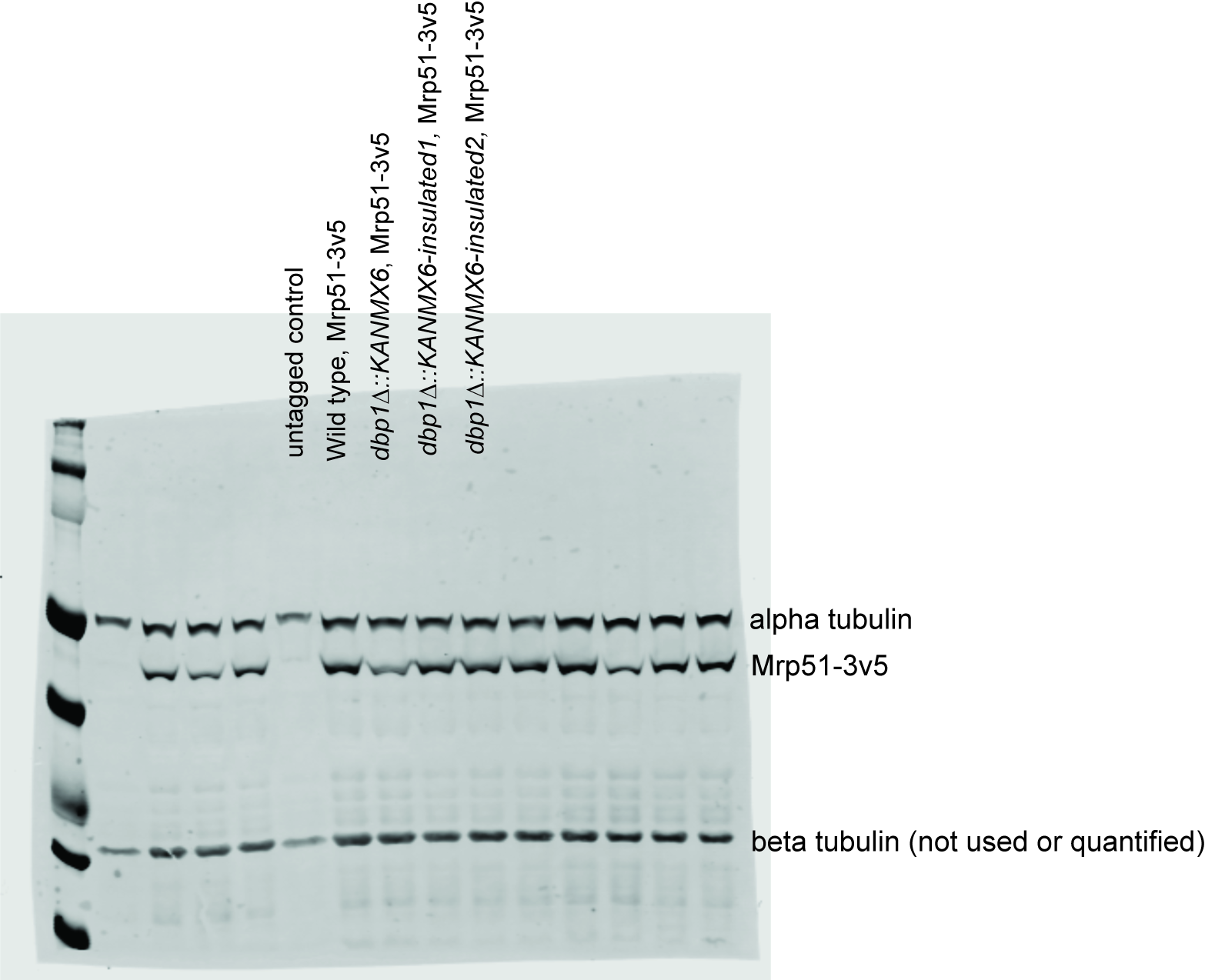

Supplement: Figure 3—source data 1. [file elife-81086-fig3-data1.zip › Figure 3 - Source Data 1/Figure 3 - Source Data 1 - labeled.tif]

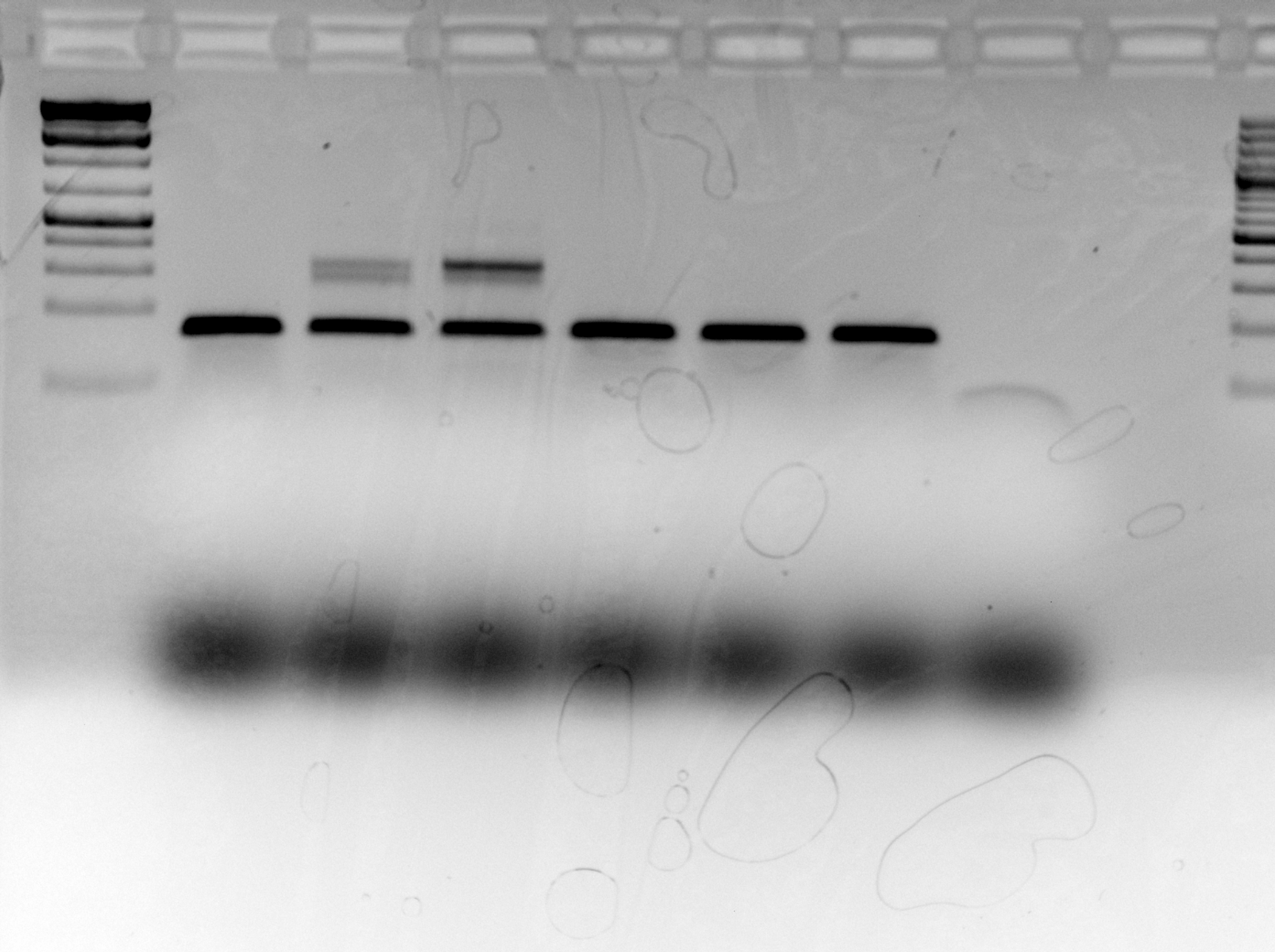

Supplement: Figure 3—figure supplement 1—source data 1. [file elife-81086-fig3-figsupp1-data1.zip › Figure 3 - figure supplement 1 - Source Data 1/Figure 3 - figure supplement 1 - Source Data 1.tif]

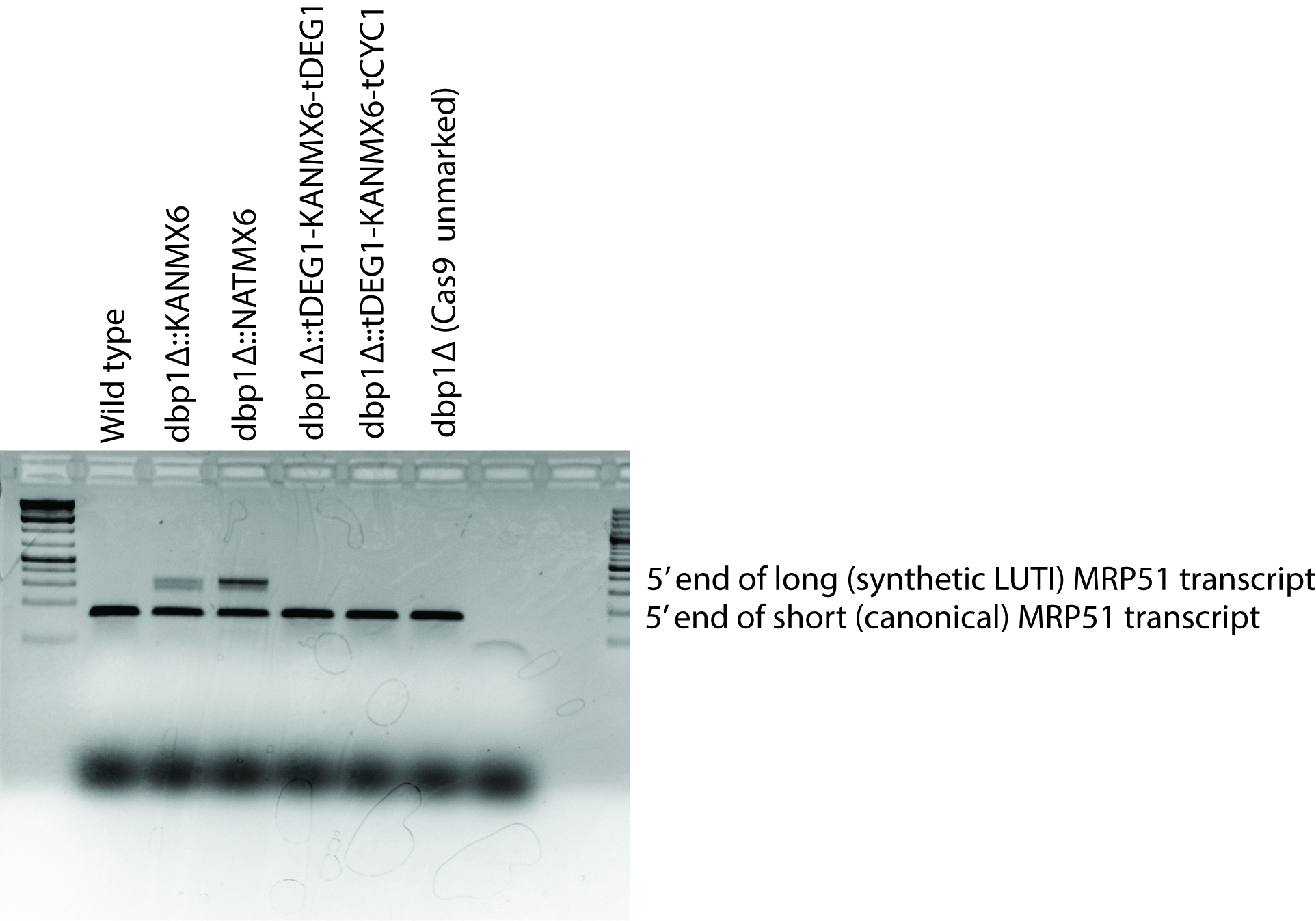

Supplement: Figure 3—figure supplement 1—source data 1. [file elife-81086-fig3-figsupp1-data1.zip › Figure 3 - figure supplement 1 - Source Data 1/Figure 3 - figure supplement 1 - Source Data 1_labeled.tif]
